# Prevention of cyclophosphamide-induced immune suppression by polysaccharides from *Apocynum venetum* flowers *via* enhancing immune response, reducing oxidative stress, and regulating gut microbiota in mice

**DOI:** 10.3389/fphar.2024.1354794

**Published:** 2024-05-23

**Authors:** Qingchun Zhao, Jinmei Wang, Haiyang Liang, Wenjing Guo, Yanhai Chu, Lijun Liu, Wenyi Kang

**Affiliations:** ^1^ National R & D Center for Edible Fungus Processing Technology, Henan University, Kaifeng, China; ^2^ College of Agriculture, Henan University, Kaifeng, China; ^3^ Joint International Research Laboratory of Food & Medicine Resource Function, Henan Province, Kaifeng, China; ^4^ Traditional Chinese Medicine Department of Huaihe Hospital, Henan University, Kaifeng, China

**Keywords:** polysaccharides, *Apocynum venetum* flowers, immune regulation, transcriptomics, intestinal flora gut microbiota, cyclophosphamide 1

## Abstract

**Introduction:**

Emerging proof suggests that *Apocynum venetum* flowers polysaccharide (AVFP) has immunomodulatory effects *in vitro*. However, the action mechanism of AVFA is still unclear *in vivo*. The purpose of this study is to probe into the potential mechanism of AVFA in immunosuppressed mice by investigating organ index, cytokine levels, anti-oxidative stress capacity, transcriptomics, and gut microbiota.

**Methods:**

Immunocompromised mice induced by cyclophosphamide (CTX) were divided into six groups. The enzyme-labeled method, hematoxylin and eosin, transcriptomics, and high-throughput sequencing were used to detect the regulatory effects of AVFP on immunocompromised mice and the function of AVFP on the concentration of short-chain fatty acids (SCFAs) by high-performance liquid chromatography (HPLC) analysis. The Spearman correlation analysis was used to analyze the correlation between the intestinal microbiota and biochemical indexes.

**Results:**

The experimental results illustrated that AVFP has protective effects against CTX-induced immunosuppression in mice by prominently increasing the organ index and levels of anti-inflammatory factors in serum in addition to enhancing the antioxidant capacity of the liver. Meanwhile, it could also signally decrease the level of pro-inflammatory cytokines in serum, the activity of transaminase in serum, and the content of free radicals in the liver, and alleviate the spleen tissue damage induced by CTX. Transcriptomics results discovered that AVFP could play a role in immune regulation by participating in the NF-*κ*B signaling pathway and regulating the immune-related genes *Bcl3*, *Hp*, *Lbp*, *Cebpd*, *Gstp2*, and *Lcn2*. Gut microbiota results illustrated that AVFP could increase the abundance of beneficial bacteria, reduce the abundance of harmful bacteria, and regulate the metabolic function of intestinal microorganisms while dramatically improving the content of SCFAs, modulating immune responses, and improving the host metabolism. The Spearman analysis further evaluated the association between intestinal microbiota and immune-related indicators.

**Conclusion:**

These findings demonstrated that AVFP could enhance the immune effects of the immunosuppressed mice and improve the body’s ability to resist oxidative stress.

## 1 Introduction

Immunosuppression can reduce the body’s ability to fight bacteria and viruses. With the increasing number of immune diseases in the world, improving immune protection has become an urgent problem to be solved. The three parts of the immune system are immune molecules, immune cells, and immune organs (Yin et al., 2021). When the body is confronted with external stimuli such as foreign infection, inflammation, and cancer, the immune system reacts by producing stress (Barabas et al., 2018). Polysaccharides have immune regulatory effects and play an active role in the growth of immune organs, the activation of immune cells, and the expression and release of immune cytokines (Wu et al., 2016). Studies have shown that polysaccharides could prompt the host to form a strong immune response, chiefly by irritating macrophages and stimulating the secretion of cytokines (Otha et al., 2016). Zhang et al. (2014) found that *Ganoderma atrum* polysaccharide could increase the phagocytosis of macrophages, notably improve the spleen and thymus indices, and strengthen the function of the body’s immunity. *Salviae miltiorrhizae* polysaccharide significantly stimulates the proliferation of splenocytes, promotes the production of interleukin-4 (IL-4) and IL-2, suppresses the secretion of interleukin-6 (IL-6) and TNF-α, and enhances the cell activity of natural killer (NK) cells and cytotoxic T lymphocytes (Wang et al., 2014). *Hericium erinaceus* polysaccharide has the same regulatory effect on TNF-α, IL-2, and IL-6 in murine macrophages (Wu and Huang, 2021).

*Apocynum venetum* belongs to the *Apocynum* family, and China is the largest distribution area of *A. venetum* in the world (Ren, Cao, Chen & Li, 2008). *Apocynum venetum* has the capacity for potent antioxidation, liver protection, and cardiotonic, lipid-lowering, and anticoagulant properties (Kobayashi et al., 2004; Grundmann et al., 2009; Ru et al., 2012). In our previous research studies, *Apocynum venetum* flowers polysaccharide (AVFP) was non-toxic, and two polysaccharides, namely, Vp2a-Ii and VP3, were isolated from *A. venetum* flowers (Wang et al., 2022). Through further *in vitro* experiments, it was concluded that Vp2a-Ii and VP3 stimulate the expression of cytokines by stimulating the NF-*κ*B/MAPK signaling pathway, and play an immunoregulatory role (Wang et al., 2019). However, there are no reports on the immune research of AVFP *in vivo*.

Transcriptome sequencing can determine changes in mRNA expression levels (Jiang et al., 2020). In recent years, transcriptome sequencing has been applied to the study of pathogenesis and the screening of biomarker indicators, which plays an important role in the diagnosis and prognosis of complex diseases such as immune diseases and diabetes (Hao et al., 2021; Roy et al., 2021). Wu et al. (2021) used RNA-Seq to study the immunoregulatory mechanism of exopolysaccharides in *Chlorella* sp. and obtained a total of 4,824 differently expressed genes (DEGs) from the results, including 2,165 upregulated and 2,659 downregulated DEGs. Mariann et al. (2020) used RNA-Seq to analyze the immunomodulatory effect of sulfated polysaccharide ulvanthe on *Senegalese sole*. Through the experiment, it was found that 402 differential genes were selected in the treated and control groups. After administration of sulfated polysaccharide ulvanthe, some immune-related genes were significantly upregulated, such as chemokines, proteasomes, and antigen presentation. The intestine, as an immune organ, plays a particularly important role in the immune response. Therefore, the gut microbiota and the immune system are in a mutually regulated and interdependent relationship (Round and Mazmanian, 2009). In recent years, more and more studies have shown that polysaccharides can safeguard the intestinal mucosa and regulate the proportion of microbiota. Polysaccharides can regulate the proportion of beneficial microbiota, inhibit the proportion of harmful microbiota, and increase the content of short-chain fatty acids (SCFAs) to enhance immunity. Bai et al. (2022) found that polysaccharides from Fuzhuan brick tea could regulate the gut microbiota by improving the relative proportion of Muribaculaceae and decreasing the relative proportion of Lachnospiraceae and Desulfovibrionaceae, and increasing the content of SCFAs in cecal contents. Chen et al. (2021) confirmed that the beneficial modulation of polysaccharides from *Fortunella margarita* can alter the levels of SCFAs. Therefore, transcriptomic techniques and gut microbiota are often used to research the regulation mechanisms of polysaccharides on the body’s immunity.

Thus, based on these research backgrounds, the aim of the current study is to investigate the effect of AVFP on cyclophosphamide (CXT)-induced immunomodulation from the perspective of the gut microbiota and its genes to provide evidence for the potential utilization of AVFP.

## 2 Materials and methods

### 2.1 Materials and reagents

CXT (20112325) was purchased from Jiangsu Hengrui Pharmaceutical Co., LTD. (Nanjing, China). Lentinus edodes polysaccharide (2011060) was purchased from Hebei Guangren Pharmaceutical Co. LTD. (Shijiazhuang, China). AST (20211115), ALT (20211115), GSH-Px (20210813), MDA (20210813), SOD (20210813), and ROS (20210813) were purchased from Nanjing Jiancheng Technology Co., LTD. (Nanjing, China). TNF-α (20211112) and IL-2 (20211112) were purchased from Suzhou Sizhengbai Biotechnology Co., LTD. (Suzhou, China). The multimode microplate reader used was from Thermo Fisher Scientific, China. We used the Illumina MiSeq/NovaSeq platform (Illumina, China) and the ultra-high-throughput genetic sequencer DNBSEQ-T7 (BGI, China).

*Apocynum venetum* flowers were collected from Aksu Region, Xinjiang, in July 2015. They were identified by Professor Changqin Li. Dry *A. venetum* flowers were soaked in petroleum ether and 70% ethanol for 3 days. After drying, distilled water was added according to a solid–liquid ratio of 1:30, and the filtrate was extracted twice at 90°C for 3 h. The filtrate was concentrated and precipitated with 70% ethanol, then deposited with alcohol at 4°C for 24 h, and then filtered and precipitated with anhydrous ethanol, acetone, and petroleum ether 2 times. Protein was removed using the Sevag method, and after re-precipitation with ethanol, the AVFP was freeze-dried to solid (Wang et al., 2019).

### 2.2 Animal model and experimental protocol

Specific pathogen-free (SPF) Kunming (KM) male mice (5–6 weeks, 18–22 g) were obtained from Henan Provincial Laboratory Animal Center (License No. SCXK (Yu) 2019-0002) and then raised for adaptation (temperature 25°C ± 2°C, light 12 h/day, humidity 40%–45%).

After 1 week of adaptation feeding, mice were randomly allocated to six groups (n = 6/group): control group, model control (Mod) group, positive control (LEP) group, AVFP high-dose (AVFP-H) group, AVFP medium-dose (AVFP-M) group, and AVFP low-dose (AVFP-L) group. According to the references, mice in the control and Mod groups were gavaged daily with distilled water, mice in the PC group were gavaged daily with 3 mg/kg *Lentinus edodes* polysaccharide tablets, and mice in the AVFP-H, AVFP-M, and AVFP-L groups were gavaged daily with 600 mg/kg, 300 mg/kg, and 150 mg/kg AVFP, respectively, for 21 days, and the gavage volume was 0.1 mL/10 g (*L. edodes* polysaccharide tablets and AVFP were dissolved using distilled water). During the last 3 days of the treatment, an intraperitoneal injection of 80 mg/kg was administered to mice in the treatment group. The above animal experimental designs are briefly listed in Table 1. Mice were weighed every other day. At the end of the experiment, the mice were anesthetized with isoflurane (3% for induced anesthesia, with an oxygen flow rate of 300–500 mL/min; 2% for sustained anesthesia, with an oxygen flow rate of 150–250 mL/min), and the isoflurane concentration was controlled by an anesthetic gas evaporator (Puxin, ZR-02, China). After deep anesthesia, blood was taken from the eyeball. The blood was left at room temperature for 2 h and then centrifuged (3500 rpm/min, 10 min), and the supernatant was taken as serum. Spleen, thymus, liver, and cecum contents were removed and kept at −80°C for subsequent assay analysis (Liang et al., 2021).

**TABLE 1:** Group name, number, and processing details.

Group	Treatment method	Number of groups
Control	Gavaging with distilled water daily	6
Mod	Gavaging with distilled water daily; intraperitoneal injection of CTX at 80 mg/kg in the last 3 days of the experiment	6
LEP	Gavaging with 3 mg/kg *Lentinus edodes* polysaccharide tablets daily; intraperitoneal injection of CTX at 80 mg/kg in the last 3 days of the experiment	6
AVFP-H	Gavaging with 600 mg/kg AVFP daily; intraperitoneal injection of CTX at 80 mg/kg in the last 3 days of the experiment	6
AVFP-M	Gavaging with 300 mg/kg AVFP daily; intraperitoneal injection of CTX at 80 mg/kg in the last 3 days of the experiment	6
AVFP-L	Gavaging with 150 mg/kg AVFP daily; intraperitoneal injection of CTX at 80 mg/kg in the last 3 days of the experiment	6

### 2.3 Organ index calculation

the organ index calculation was performed using the following formula: organ index = immune organ weight (mg)/body weight (10 g).

### 2.4 Cytokine detection in serum

The serum stored at −80°C was taken and placed at room temperature. Blank and sample wells were set up separately. The contents of tumor necrosis factor *α* (TNF-α), reactive oxygen species (ROS), cytokines, and interleukin 2 (IL-2) were detected by enzyme-linked immunoassay according to the manufacturer’s kit instructions.

### 2.5 Detection of aspartate aminotransferase (AST) and alanine aminotransferase (ALT) activity in serum

AST catalyzes the transamination of α-ketoglutarate and aspartate to form glutamate and oxaloacetate. It appears red–brown in alkaline solutions, and the change in absorbance is detected using a multimode microplate reader. ALT acts on a substrate composed of alanine and *α*-ketoglutarate under conditions of 37°C and pH 7.4 to form pyruvate and glutamate. A series of reactions ensue, resulting in the formation of a red–brown color, and the change in absorbance is measured. The specific experimental operation details are based on the manufacturer’s kit instructions.

### 2.6 Detection of antioxidant stress capacity in the liver

The mouse liver tissues were accurately weighed, rinsed with ice-cold saline to remove blood, and wiped dry with filter paper. Tissue homogenates were prepared at 1:9 on the basis of the instructions provided. The supernatant was collected to detect the content of superoxide dismutase (SOD), glutathione peroxidase (GSH-Px), and malondialdehyde (MDA) in mouse liver tissue.

### 2.7 Histopathological section observation

To fix approximately 1 cm of spleen tissue, it was immersed in a 4% paraformaldehyde solution for 24 h. Following fixation, trimming, dehydration, embedding, sectioning, and staining, the tissue was mounted. The Eclipse Ci-L camera microscope was used to capture images at ×200 magnification. During imaging, it was ensured that the tissue filled the entire field of view, and consistent backlighting was maintained for each photograph (Liang et al., 2022).

### 2.8 Transcriptomics research

Transcriptomic analysis was performed on nine spleen samples from the control, Mod, and AVFP-M groups. First, we took 60 mg of tissue and pulverized it into powder in liquid nitrogen. The sample was centrifuged after homogenizing. We added 0.3 mL of isoamyl alcohol to the supernatant and centrifuged the sample. After that, 1 mL of 75% ethanol was added to wash twice, and the sample was centrifuged. In the end, DEPC-treated water was added to dissolve RNA, followed by the identification and quantification of total RNA using a NanoDrop spectrophotometer and the Agilent 2100 Bioanalyzer. Quality control was carried out on the data obtained by sequencing. After passing the quality control, gene quantitative analysis and gene screening based on the gene expression level were carried out. According to the differential genes, GO functional significance enrichment analysis, pathway significance enrichment analysis, and other further mining analyses were carried out.

### 2.9 Intestinal flora detection

Three cecal contents were selected from each group for DNA extraction, PCR amplification, product purification, library preparation, and detection, and multiple libraries were mixed for Illumina MiSeq/NovaSeq sequencing. The 16S rDNA amplification sequencing for this study was completed by Suzhou Genomics Co., Ltd. (Jiang et al., 2020).

### 2.10 SCFA detection

A total of 0.3 g of cecal contents were taken from each mouse, 1 mL of deionized water was added, and centrifugation was performed at 12,000 rpm/min to obtain the supernatant, and then HCL and ether were added sequentially for extraction. The mixture was passed through a 0.22-um filter membrane. SCFAs in cecal contents were detected through high-performance liquid chromatography (HPLC) (Liang et al., 2022).

### 2.11 Statistical analysis

The data were evaluated using the Shapiro–Wilk test to confirm the normality of the distribution. All data were shown as the means ± standard error. SPSS 26.0 software was used to carry out data statistics. A one-way ANOVA and Dunnett’s *post hoc* multiple comparison tests were conducted. *p* < 0.05 was considered significant.

## 3 Results

### 3.1 AVFP increased the body weight and organ index and regulated serum cytokine levels in CTX-induced mice

**FIGURE 1:**
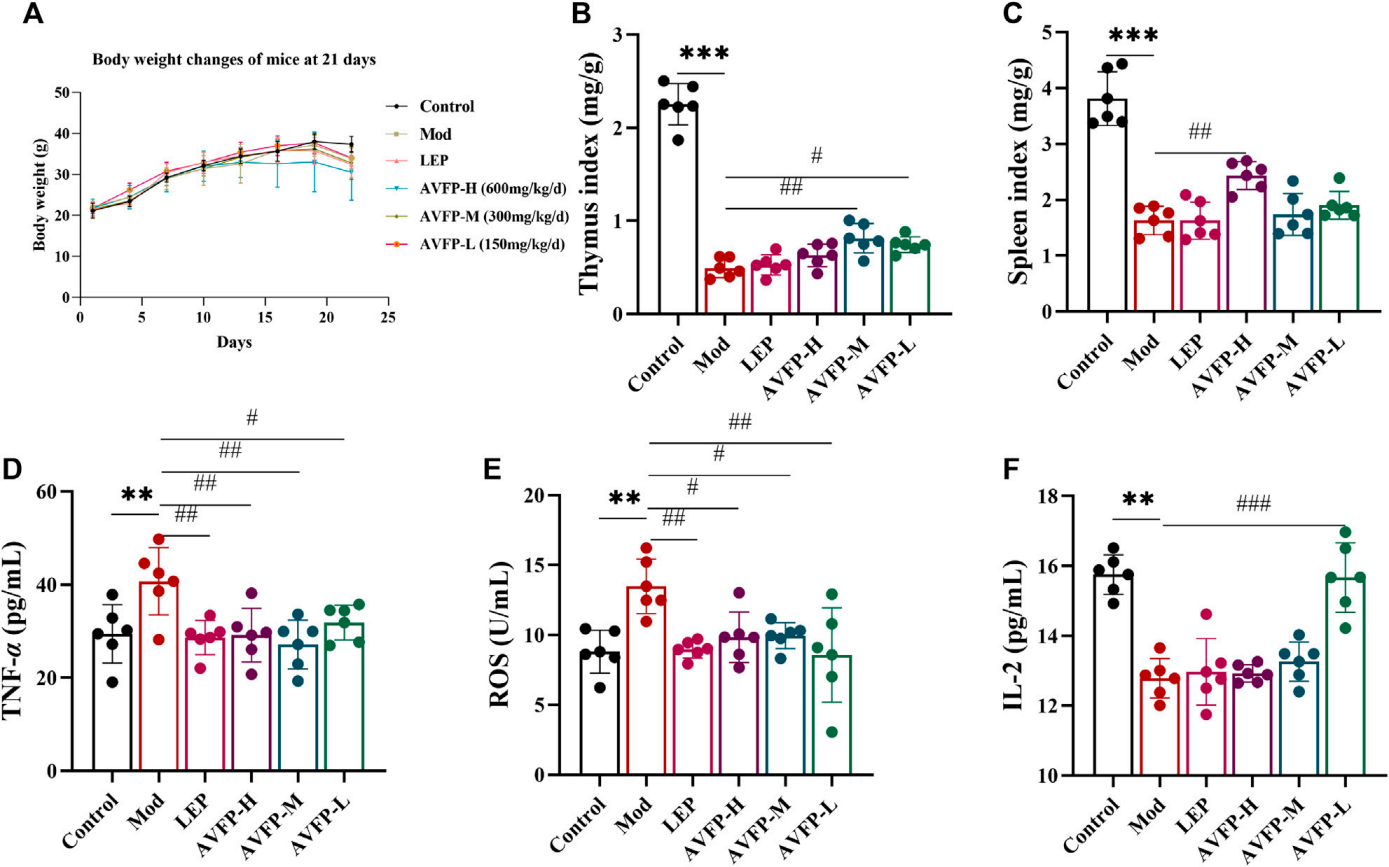
Effects of AVFP on body weight gain **(A)**, thymus index **(B)**, spleen index **(C)**, TNF-α **(D)**, ROS **(E)**, and IL-2 **(F)** in mice. Mod *versus* control: ***p < 0.001, **p < 0.01, *p < 0.05; ^###^ *p* < 0.001, ^##^ *p* < 0.01, ^#^ *p* < 0.05 *versus* Mod (n = 6), one-way ANOVA.

Body weight is closely related to the number and function of immune cells, and the thymus and spleen are essential immune organs for lymphocyte differentiation, maturation, and immune responses. To evaluate the effect of AVFP on immunosuppressed mice, indicators such as the body weight, immune organ index, and cytokine secretion capacity of the mice were measured. We monitored the weight of the mice every other day. AVFP ameliorated the loss of body weight in CTX-induced mice (Figure 1A). In addition, the thymus index and spleen index were extremely reduced in CTX-induced mice. After the AVFP gavage, the thymus index in the AVFP-M and AVFP-L groups was particularly increased, and the spleen index in the AVFP-H group was dramatically increased (Figures 1B, C). In Figures 1D–F, the levels of TNF-α and ROS in the Mod group were higher than those in the control group, suggesting that CTX could induce immunosuppression in mice. The levels of TNF-α and ROS were reduced by AVFP gavage, so the levels in the AVFP-H, AVFP-M, and AVFP-L groups were significantly reduced. On the contrary, the levels of IL-2 were remarkably reduced in the Mod group, illustrating that CTX could restrain the immune capacity in mice. After treatment, the levels of IL-2 significantly increased in the AVFP-L group. These findings demonstrated that AVFP could improve weight loss in immunosuppressed mice by CTX induction, improve the protective ability of immune organs, ameliorate the level of cytokines, and increase the immune capacity of the body.

### 3.2 AVFP reduced AST and ALT activities and enhanced the antioxidant capacity in CTX-induced mice

**FIGURE 2:**
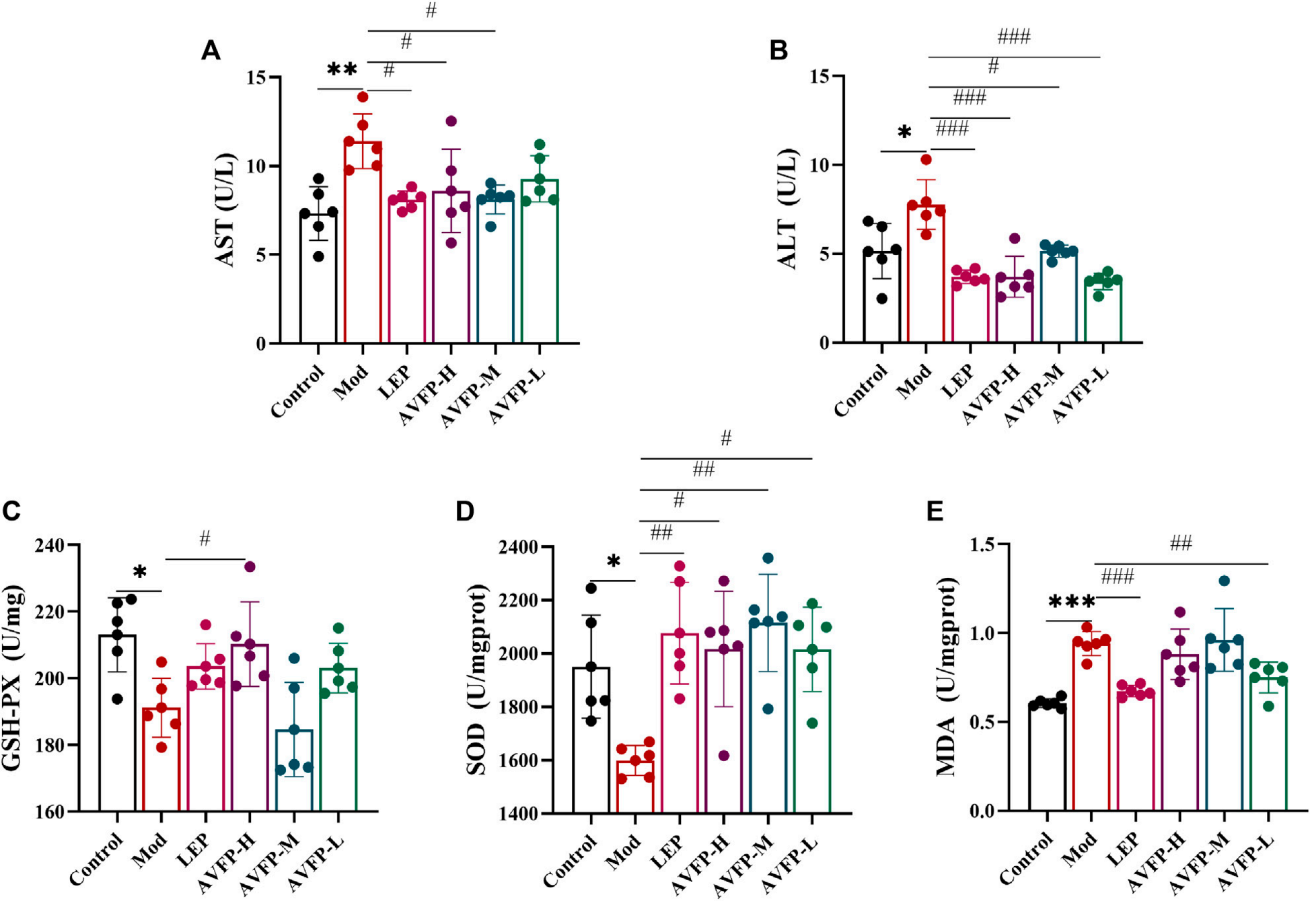
Effects of AVFP on AST **(A)**, ALT **(B)**, GSH-Px **(C)**, SOD **(D)**, and MDA **(E)** in mice. Mod *versus* control: ***p < 0.001, **p < 0.01, *p < 0.05; ^###^ *p* < 0.001, ^##^ *p* < 0.01, ^#^ *p* < 0.05 *versus* Mod (n = 6), one-way ANOVA.

CTX causes immunosuppression and also generates free radicals in the body, leading to oxidative stress and damaging liver function (Jing et al., 2018). When inflammation occurs in liver cells, they become damaged, and transaminases are released into the blood, resulting in an increase in serum transaminase levels. We tested the antioxidant stress capacity and transaminase activity of mice. In Figures 2A, B, AST, and ALT activities in the Mod group were significantly improved compared with those in the control group. After AVFP gavage, AST activity in the LEP, AVFP-H, and AVFP-M groups was significantly reduced, and ALT activity in both LEP and AVFP gavage groups was remarkably decreased, especially in the polysaccharide-administered groups. As illustrated in Figures 2C, D, CTX could significantly reduce the activities of GSH-Px and SOD, increase the activity of MDA, and reduce the ability to resist oxidative stress. However, after AVFP gavage, GSH-Px and SOD activities were significantly increased. However, the content of MDA was extremely decreased in the LEP and AVFP-L groups, as shown in Figure 2E. Thus, AVFP could decrease AST and ALT activities and enhance the body’s ability to resist oxidative stress.

### 3.3 AVFP prevented histopathological changes in spleen tissue in CTX-induced mice

**FIGURE 3:**
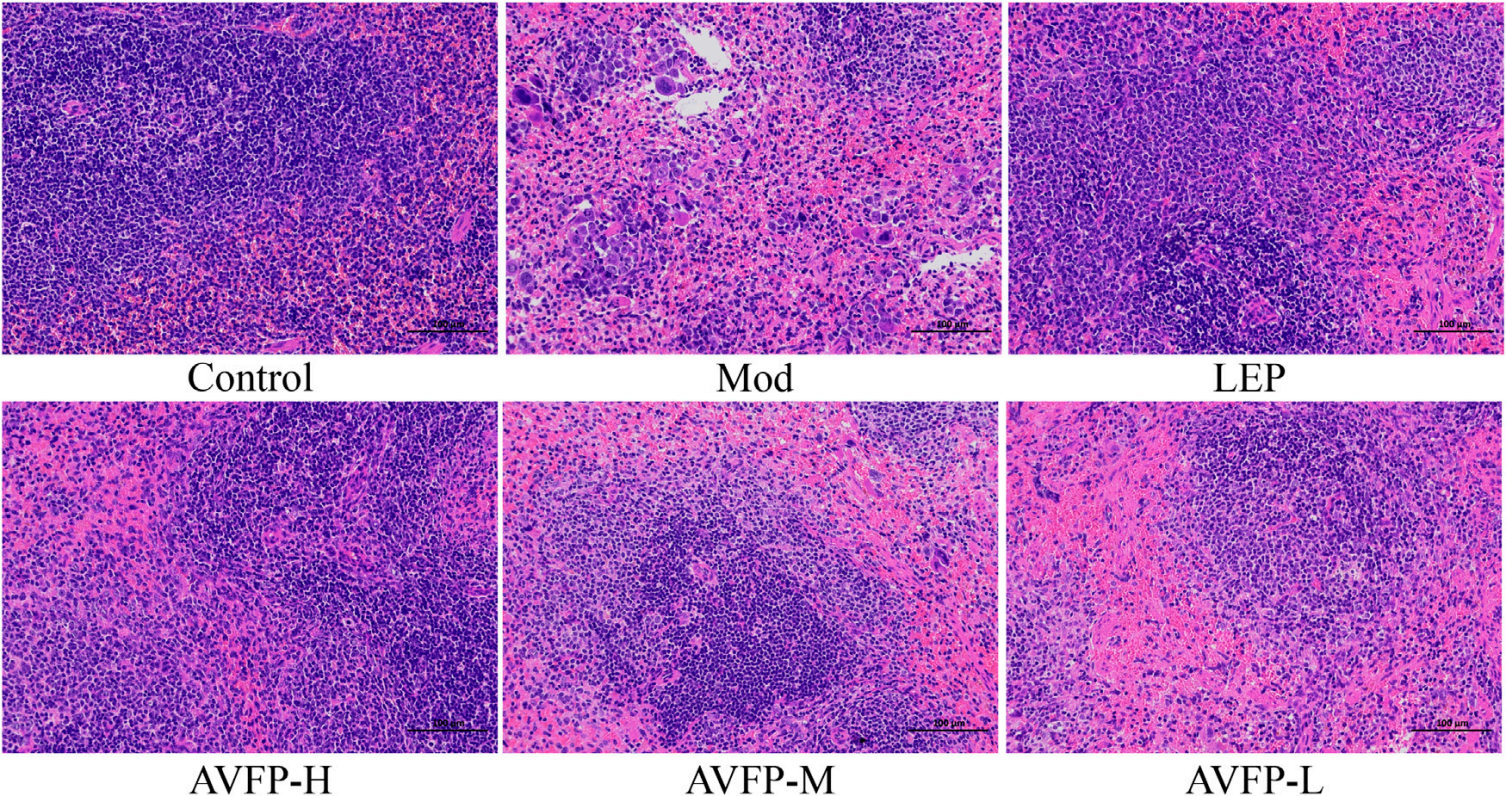
Effect of AVFP on spleen organ in mice.

The spleen is an important immune organ, and the state of CTX-induced immunocompromised mice can be judged by observing and photographing the histological morphology of the spleen (Li et al., 2017). As shown in Figure 3, the pathological section observation showed that the spleen of mice in the control group had normal morphology and neatly arranged cells. After CTX modeling, the area of the white pulp was smaller, the proportion of the red pulp was larger, and the boundary between the white pulp and the red pulp was not obvious. After the administration of AVFP, the pathological recovery degree of the white pulp in the AVFP-H group was similar to that in the LEP group, but there was no significant change in the AVFP-M and AVFP-L groups. Our experimental results showed that AVFP could reduce CTX-induced spleen damage, protect immune organs, and improve the body’s immune stress ability.

### 3.4 Gene identification and differential gene screening

**FIGURE 4:**
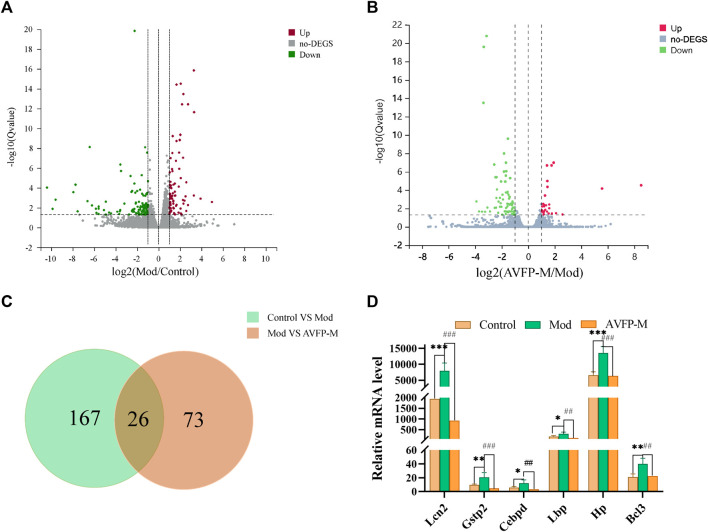
Volcano plots **(A, B)** and differential genes Venn **(C)** of Mod vs. control and AVFP-H vs. Mod, and expression levels of key genes in control, Mod, and AVFP-M groups **(D)**. Mod *versus* control: ***p < 0.001, **p < 0.01, *p < 0.05; ^###^ *p* < 0.001, ^##^ *p* < 0.01 *versus* Mod (n = 3).

By sequencing the RNA of different samples, transcriptomics allows for the comparison of gene expression levels and changes between study samples, thereby further exploring the mechanisms underlying the occurrence of disease. A total of nine samples were detected *via* the RNA-Seq platform. The average comparison rate of the sample comparison genome was 92.30%, and the average comparison rate of the comparison gene set was 71.27%. A total of 16,623 genes were detected. Differential genes were screened with |log2FC| >1 and *p* < 0.05. A total of 193 differential genes were identified in the control and Mod groups, and 99 differential genes were identified in the Mod and AVFP-M groups. Compared with the control group, 82 differential genes were upregulated, and 111 differential genes were downregulated in the Mod group. Compared with the Mod group, 28 differential genes were upregulated, and 71 differential genes were downregulated in the AVFP-M group, as illustrated in Figures 4A, B. Combined with the literature, the 26 differential genes shared by the three groups were further screened (Figure 4C), and finally, 6 differential genes with greater immune-related and antioxidant correlation were obtained, namely, *Bcl3*, *Hp*, *Lbp*, *Cebpd*, *Gstp2*, and *Lcn2*. The expression levels derived from transcriptomics are shown in Figure 4D.

### 3.5 Differential gene GO functional enrichment analysis and KEGG pathway enrichment analysis

**FIGURE 5:**
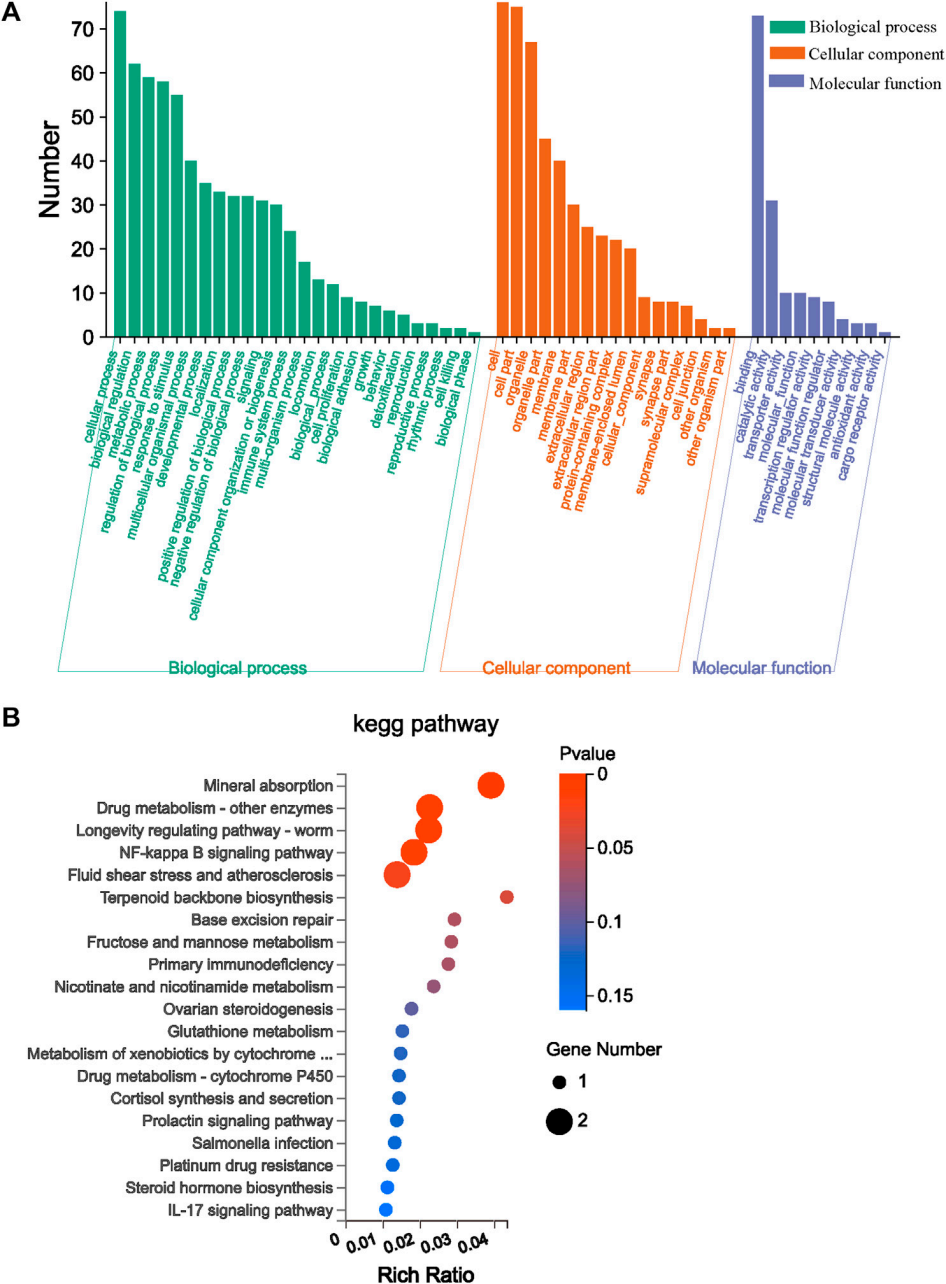
Effect of AVFP by GO function enrichment analysis of major differentially expressed genes **(A)** and dramatically enriched KEGG pathway **(B)**.

GO and KEGG enrichment analyses play significant roles in bioinformatics research. They can assist researchers gain a deeper understanding of the functions, pathways, and biological processes of gene sets under specific conditions. In Figure 5A, 99 differential genes between the Mod group and AVFP-M group are annotated into 53 GO items: 26 biological processes, 17 cellular components, and 10 molecular functions, among which the immune system process, antioxidant activity, and detoxification were related to immunity. The KEGG enrichment analysis is helpful to further research the biological function of the genes. A total of 26 DEGs into 43 KEGG pathways were enriched, of which the first 20 KEGG pathways are shown in Figure 5B. The abscissa is obtained by counting the number of differential genes in the pathway. In Figure 5B, the higher the rich factor value, the higher the relative percentage of differential genes in the pathway. Differential genes were enriched by immune-related signaling pathways, such as the NF-*κ*B signaling pathway (*p* = 0.013).

### 3.6 AVFP regulated the gut microbiota in CTX-induced mice

**FIGURE 6:**
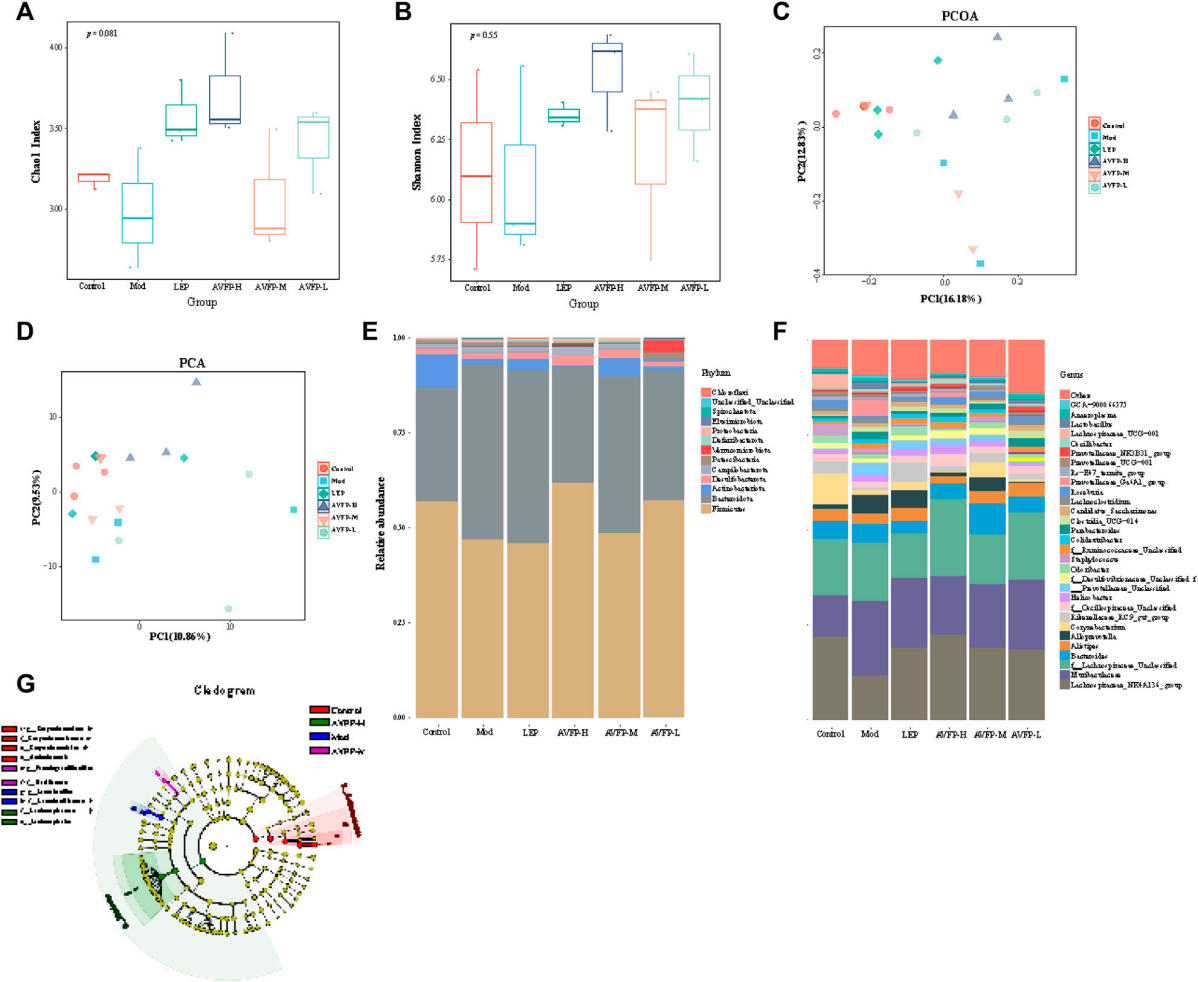
Effect of AVFP on α-diversity **(A, B)** and β-diversity **(C, D)** in mice. Effect of AVFP on relative abundance of dominant groups at phylum **(E)** and genus **(F)** level in mice. **(G)** Lefse analyze (n = 3).

Plant polysaccharides also have the effect of regulating the structure of the gut microbiota and improving intestinal microecology. We used 16S rDNA technology to evaluate the impact of AVFP on the gut microbiota of immunocompromised mice. In Figure 6A, the Chao1 index of the LEP, AVFP-H, and AVFP-L groups was higher than that of the Mod group, suggesting that AVFP could elevate the diversity of gut microbiota in CTX-induced mice. In Figure 6B, the Shannon index was considerably improved in the AVFP-H, AVFP-M, AVFP-L, and LEP groups, suggesting that AVFP could enhance the diversity of intestinal flora in mice. *β*-diversity is used to analyze the diversity difference between different samples, and PCA can analyze the distribution of sample colonies based on the distance of the samples. In Figures 6C, D, it can be seen that the distance between the control group and the LEP group was close, and the difference was small. Likewise, mice treated with AVFP were remarkably different from CXT-induced mice in terms of gut microbiota distribution and similar to the control group, indicating that AVFP could regulate the structural composition of intestinal microbes in CTX-induced mice.

In Figure 6E, at the phylum level, AVFP could ameliorate the CTX-induced increase of *Bacteroidota* abundance and decrease of *Firmicutes* and *Proteobacteria* abundance*.* At the genus level (Figure 6F), compared to the control group, the abundance of *Helicobacter*, *Alloprevotella*, Muribaculaceae, *Parabacteroides*, *Lachnoclostridium*, *Blautia*, Prevotellaceae_Ga6A1_group, and *Lactobacillus* was remarkably enriched in CTX-induced mice. However, the CTX-increased abundance of *Alloprevotella*, Muribaculaceae, *Parabacteroides*, *Lachnoclostridium*, Prevotellaceae_Ga6A1_group, and *Lactobacillus* was reduced by AVFP gavage. Similarly, CTX decreased the abundance of Lachnospiraceae_NK4A136_group, *Roseburia*, Lachnospiraceae_UCG-001, *Corynebacterium*, and AVFP could reverse these changes. LEfSe analysis also verified the microbiota abundance at the genus level (Figure 6G).

Collectively, the CTX-induced gut dysbiosis was restored *by* AVFP gavage.

### 3.7 AVFP altered metabolic functions and colony SCFAs levels in CTX-induced mice

**FIGURE 7:**
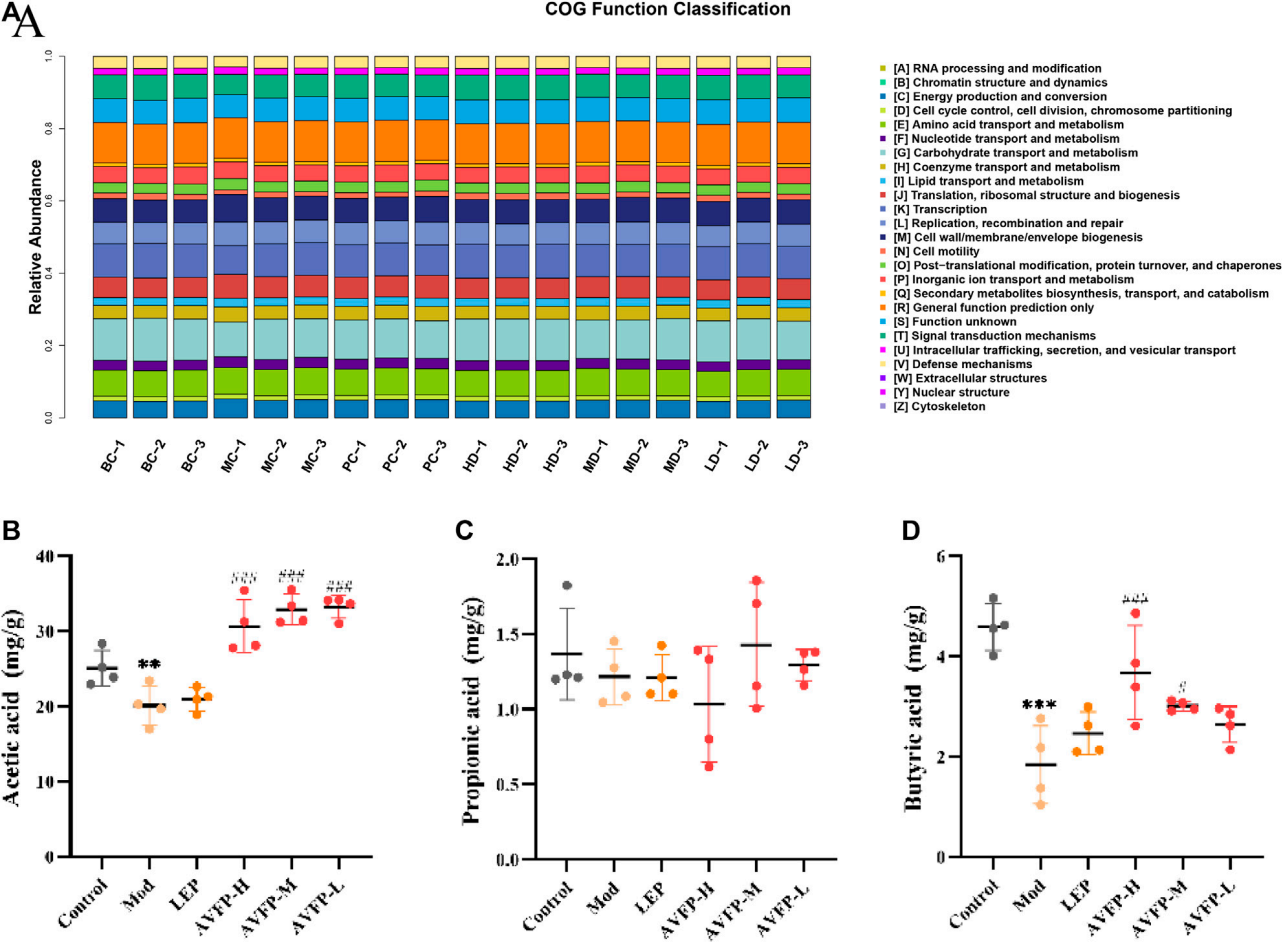
Distribution of COG functional abundance. **(A)** Effect of AVFP on SCFAs in mice **(** **B–D** **)**. Mod *versus* control: ^***^ *p* < 0.001, ^**^ *p* < 0.05; ^###^ *p* < 0.001, ^#^ *p* < 0.05 *versus* Mod (n = 4), one-way ANOVA.

Likewise, the COG pathway was used to evaluate the potential function of the gut microbiota. AVFP mainly reversed 10 metabolic pathways, namely, chromatin structure and dynamics, amino acid transport and metabolism, nucleotide transport, and metabolism, coenzyme transport and metabolism, transcription, cell wall/membrane/envelope, cell motility, and secondary metabolite biosynthesis, transport, and catabolism (Figure 7A). The results indicated that AVFP could enhance the immunity of the body by regulating the metabolic function of intestinal microorganisms.

In Figures 7B–D, CTX could evidently reduce the levels of acetic acid and butyric acid but had no apparent effect on propionic acid. After the administration of AVFP, the levels of acetic acid and butyric acid were elevated in the AVFP-H and AVFP-M groups, but the levels of butyric acid in the AVFP-L group did not change significantly. However, AVFP also had no overt effect on the levels of propionic acid. This implied that AVFP could regulate the gut microbiota by improving the levels of acetic acid and butyric acid and enhancing the effect of immunity.

### 3.8 Correlation among cytokines, biochemical indices, and gut microbiota

**FIGURE 8:**
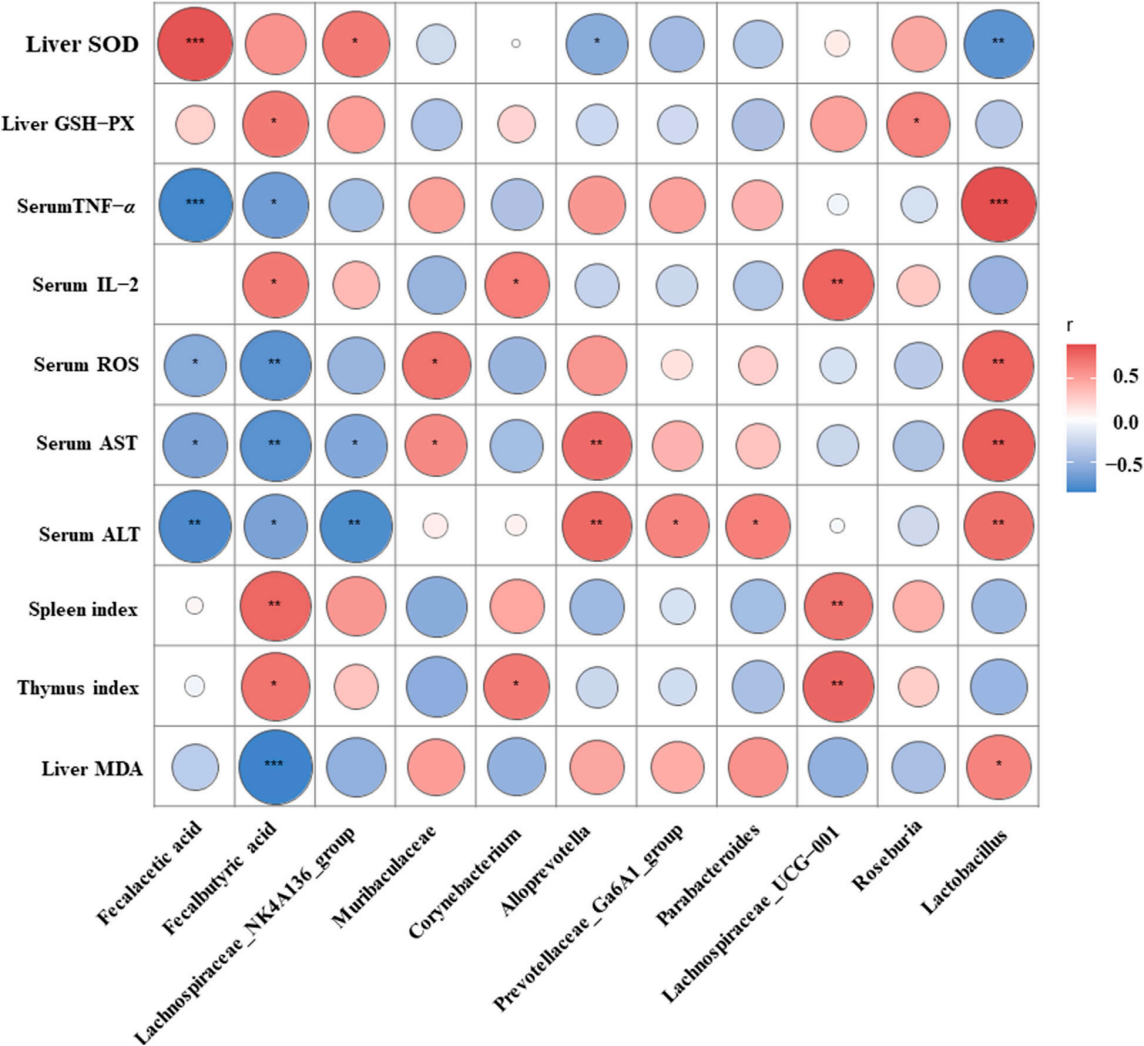
Spearman’s correlation heatmap between cytokines, biochemical indices, and gut microbiota. Significance representation method: *p < 0.05, **p < 0.01, and ***p < 0.001. Red, positive correlation; blue, negative correlation.

To further explore the potential relationship among gut microbiota, biochemical markers, and immunity *via* the Spearman correlation analysis (Figure 8), fecal SCFA levels, Lachnospiraceae_NK4A136_group, *Corynebacterium*, Lachnospiraceae_UCG-001, and *Roseburia* were negatively connected with serum TNF-α, serum ROS, serum ALT, serum AST, and liver MDA, and positively related with liver SOD, liver GSH-Px, serum IL-2, and the thymus and spleen indexes. Conversely, the abundance of Muribaculaceae, *Alloprevotella*, Prevotellaceae_Ga6A1_group, *Parabacteroides*, and *Lactobacillus* was positively associated with liver MDA, serum ALT, serum AST, and serum TNF-α, and negatively associated with liver SOD, liver GSH-Px, serum IL-2, and the thymus and spleen indexes. These results demonstrated that the gut microbiota could modulate immune-related markers to ameliorate CTX-induced immunodeficiency.

## 4 Discussion

CTX could weaken the normal body weight in mice. It was found that the weight of the LEP, AVFP-M, and AVFP-L groups was comparatively stable compared with the Mod group, indicating that AVFP could ameliorate the loss of body weight in CTX-induced mice. The thymus and spleen are the most vital immune defense systems of the body (Sun et al., 2016). Studies show that most polysaccharides promote the spleen and thymus indexes in CTX-induced mice (Li et al., 2017). Our data suggested that AVFP could alleviate the organ damage caused by CTX and improve the organ index, which is in accordance with the study by Chu et al. (2020), who showed that *Apios americana* Medik flower polysaccharide could strikingly improve the organ indices of immunosuppressed mice induced *via* CTX.

Most polysaccharides can regulate the production of small-molecule bioactive substances, such as cytokines, in immune responses (Aringer and Smolen, 2005). They are released into the bloodstream and act on specific target organs. Liang et al. (2022) showed that *Nigella sativa* seed polysaccharide enhances immunity *by reducing* TNF-α secretion in immunosuppressed mice. TNF-α is a pro-inflammatory cytokine mainly produced by macrophages. When the body is stimulated by the outside environment, it can participate in normal immune responses (Mercado et al., 2012). As a by-product of oxidative phosphorylation, ROS plays a particularly important role in maintaining the immune response in immune organs (Lessard et al., 2015). Liu et al. (2018) showed that *Antrodia cinnamomea* polysaccharides evidently improved the secretion of IL-2 to enhance body immunity in CTX-induced mice. IL-2, mainly produced by activated T cells, promotes the growth, proliferation, and differentiation of lymphocytes and activates macrophages (Lopes et al., 2020). In this study, AVFP could obviously reduce the content of ROS and TNF-αand significantly increase the content of IL-2, indicating that AVFP could improve the level of cytokines and improve the body’s immune response.

CTX can cause liver damage, reduce the activity of antioxidant enzymes, cause oxidative stress, and generate large amounts of ROS (Tong et al., 2017), which could cause oxidative damage to immune organs and further damage the immune function of the human body. Excessive ROS leads to elevated levels of MDA (Guo et al., 2022). The content of MDA indirectly reflects the degree of damage caused by free radicals. SOD and GSH-Px could eliminate oxygen free radicals and are generally considered to be indicators of the body’s antioxidant defense system. Liu et al. (2018) found that *A. cinnamomea* polysaccharides effectively increased the total antioxidant capacity of the body by stimulating the activity of antioxidant enzymes in the spleen and by inhibiting the increase in ROS and MDA levels. Niu et al. (2020) also found that *Malus halliana* flower polysaccharides increased the vitality of SOD and CAT levels in the spleen, reduced the content of MDA, and improved CTX-induced oxidative stress. In this experiment, compared with the Mod group, AVFP could significantly increase the levels of GSH-Px and SOD. At the same time, the content of MDA in the AVFP-L group was also significantly decreased compared with the Mod group. The results indicated that AVFP could improve the vitality of GSH-Px and SOD and reduce the content of MDA to improve the body’s ability to resist oxidative stress.

Transcriptome sequencing could comprehensively reveal the global gene expression of individual organisms at specific times and tissues (Wang et al., 2009). The regulation of the transcriptional level is the most in-depth research and the most important regulation method for organisms. Chen et al. (2022) studied the effect of *β*-glucan on the immune indices of *Epinephelus fuscoguttatus* using RNA-Seq technology. A total of 8,014 differential genes were screened out, and the expression levels of IL-6 and TIRAP genes were found to be higher. Through KEGG enrichment analysis, JAK-STAT and Toll-like receptor signaling pathways were found to be associated with immunity. In this study, the gene expression analysis of 9 samples from the control, Mod, and AVFP-M groups of mice livers was carried out using RNA-Seq, and 16,623 genes were detected. However, 193 differential genes were screened by the control and Mod groups, and 99 differential genes were screened by the Mod and AVFP-M groups. Six genes with strong immune-related correlations were obtained through further screening of expression levels and the literature, including *Bcl3*, *Hp*, *Lbp*, *Cebpd*, and *Lcn2*. Through further analysis with GO and KEGG, it was found that the differential genes were mainly related to the immune system process and detoxification immune function, and the most abundant immune signaling pathway of the differential genes was the NF-*κ*B signaling pathway. These differential genes may be involved in the immune mechanism by regulating the secretion of cytokines and the NF-*κ*B signaling pathway. For example, *Bcl3* regulates the activity of the NF-*κ*B pathway and exerts pro- and anti-inflammatory functions by binding to different subunits. *Bcl3* can upregulate the expression of IL-6 and TNF-α in mouse liver and promote the occurrence of inflammation (Zhang et al., 2021). *Hp* is a heme-iron chelator synthesized primarily in the liver, and *Hp* expression is regulated by a variety of cellular products and cellular conditions, including bacterial endotoxins and pro-inflammatory cytokines produced by macrophage expression (Graves and Vigerust, 2016). *Lbp*, an acute inflammatory response protein synthesized by the liver, can activate cells to produce cytokines in the face of various microbial ligands (Finberg et al., 2004). *Cebpd* is an important player in the inflammatory response and could be activated by inflammatory factors, including IL-6, endotoxin, and TNF-α, to regulate the function of macrophages, thereby enhancing the inflammatory response (Slofstra et al., 2007; Ko et al., 2015). *Lcn2* is a novel immune-related gene belonging to the lipid hormone family. *Lcn2* can be involved in the innate immune response and inflammatory tumor microenvironment and promote the malignant development of various cancer types (Yang et al., 2013). Through transcriptomics, it was found that AVFP can not only regulate immune-related genes but also regulate the expression of antioxidant-related genes. *Gstp2* removes ROS *via* glutathione and plays a crucial antioxidant role in the antioxidant system (Dong et al., 2013). It is concluded that AVFP could reduce the expression of *Bcl3* and *Lbp* and the secretion of TNF-α, thereby reducing the expression of *Hp* and *Cebpd*, inhibiting the occurrence of inflammation, and enhancing immunity.

Disturbed gut microbiota ratios affect physiological processes, such as oxidative stress, immune regulation, and energy metabolism (Gao et al., 2022). In this study, AVFP could regulate the imbalance and structure of the intestinal microbiota in CTX-induced immunosuppressed mice. At the phylum level, AVFP could improve the abundance of *Firmicutes*, *Actinobacteriota*, and *Desulfobacterota* and decrease the abundance of *Bacteroidota* and *Proteobacteria*, which is in accord with the study by Liang et al. (2022). *Firmicutes* and *Bacteroidota* are the two dominant bacterial groups in the gut, accounting for almost 90% of the total number of gut microbes (Tremaroli and Bäckhe, 2012). *Firmicutes* and *Bacteroidetes* have important roles in immune responses and produce SCFAs (Stojanov et al., 2020). *Proteobacteria* can promote inflammation (Hao et al., 2021), and AVFP can reduce the increase of CTX-induced *Proteobacteria* levels and improve the body’s immunity. At the genus level, AVFP could increase the abundance of beneficial bacteria, such as Lachnospiraceae_UCG-001, *Corynebacterium*, *Roseburia*, and Lachnospiraceae_NK4A136_group, and reduce the abundance of harmful bacteria, such as Muribaculaceae*, Parabacteroides*, Prevotellaceae_Ga6A1_group, and *Lactobacillus*. Lachnospiraceae_NK4A136_group produces SCFAs and suppresses inflammation (Wang et al., 2019). *Roseburia* is the main bacterium that produces butyrate, which promotes anti-inflammatory effects and enhances intestinal epithelial barrier function by irritating SCFA receptors (Fan et al., 2021; Zeng et al., 2021). At the same time, the SCFA analysis showed that AVFP could significantly increase the content of acetic acid and butyric acid. Acetic acid is a main metabolite of most bacterial fermentation and is one of the main sources of energy for the body to obtain from carbohydrates that cannot be absorbed (Koh et al., 2016). Butyric acid can promote intestinal mucosa repair and functional recovery and inhibit the formation of inflammatory cytokines, thereby having an anti-inflammatory effect. Butyric acid, as the main source of energy for the cecum, can also regulate gene expression and maintain intestinal homeostasis (Hosseini et al., 2011). Furthermore, the Spearman correlation analysis proved the regulation of gut microbiota on immune-related indicators and antioxidants.

## 5 Conclusion

In conclusion, AVFP can protect the immune organs of CTX-induced mice, regulate the levels of serum cytokines, enhance the liver’s antioxidant capacity, and decrease the activity of transaminases in the blood, which indicates that AVFP could dramatically enhance immunity and improve the body’s ability to resist oxidative stress. Furthermore, genomics showed that AVFP may improve the expression of immune-related genes *Bcl3*, *Hp*, *Lbp*, *Cebpd*, and *Lcn2* and the antioxidant-related gene *Gstp2*, and participate in the NF-*κ*B signaling pathway, immune system process, antioxidant activity, and detoxification. Meanwhile, AVFP may increase the abundance of beneficial microbiota, improve the proportion of bacteria, increase the content of intestinal SCFAs, and facilitate the steady state of the intestinal environment. Our experimental data illustrate that AVFP has potential immune-modulatory effects and antioxidant action *in vivo* and provide a theoretical basis for further research.

## Data Availability

The original contributions presented in the study are publicly available. The raw data can be found here: https://www.ncbi.nlm.nih.gov/geo/query/acc.cgi?acc=GSE267244. Further inquiries can be directed to the corresponding authors.
